# Plant Leaves Extract Irrigation on Wound Healing in Diabetic Foot Ulcers

**DOI:** 10.1155/2021/9924725

**Published:** 2021-05-11

**Authors:** Muthu Srinivasan Jayalakshmi, P. Thenmozhi, R. Vijayaraghavan

**Affiliations:** ^1^Saveetha Institute of Medical and Technical Sciences, Chennai, India; ^2^Saveetha College of Nursing SIMATS, Chennai, India

## Abstract

**Purpose:**

We aimed to evaluate and compare the efficacy of neem leaves extract with normal saline irrigation practice in wound dressing on healing outcome and clinic-physiological parameters among individuals with diabetic foot ulcer (DFU).

**Methods:**

A quasiexperimental with repeated measures design was used on two study groups. One group received neem leaf extract wound irrigation and another group received normal saline wound irrigation. Study participants were randomly assigned to each group from 100 DFU individuals. Demographic and clinical data sheets were used to collect baseline information. Random blood sugar and HbA1C measurement was performed on the initial day of visit for all participants. We used the PUSH tool for wound assessment (wound surface area, exudate amount, and tissue type), and clinic physiological parameters (temperature, pulse rate, respiration, blood pressure, wound pain, wound infection, and local warmth) assessment was performed at baseline and then at the end of each week till four weeks were completed. Participants attended a foot clinic every 3rd or 4th day for wound care.

**Results:**

Reduction of wound healing score (PUSH score) and other wound variables improved significantly in the neem leaves extract group (*p* < 0.001). There were no significant changes in the clinic-physiological parameters.

**Conclusion:**

Neem leaves extract irrigation for foot ulcers is considered to be very safe as it did not cause any complication systematically during the study. Neem leaf extract solution can be used as an alternative solution for normal saline. Managing DFU requires continuous foot care and early risk identification of ulcer.

## 1. Introduction

Most of the people with diabetes are at great risk of developing diabetic foot ulcer (DFU). The burden of ulcers is massive, leading to economic, social, and pressure on public health workers globally [1]. The reported worldwide prevalence of DFU was 6.3% in the year 2018. The occurrence of DFU is common in elderly people [2]. Prevalence is higher for men than for women. Similarly, it is higher among people with type 2 diabetes, compared with type 1 diabetes [3]. Studies have shown that above 5% of diabetics were proven to have foot ulceration, whereas 15% have a lifetime risk of foot complication [4]. Some reasons include lack of awareness, late reports by individuals, walking barefoot, low socioeconomic status, and lack of knowledge about diabetic foot care [5]. Risk factors of foot ulceration vary from person to person, but some of them are common. Peripheral neuropathy, peripheral arterial disease, poor glucose control, poor footwear, underlying infection and duration of diabetes, cigarette smoking, and diabetic nephropathy are recognized risk factors for foot ulceration. Patients with peripheral neuropathy are at risk of developing neuropathic ulcers [6]. DFU can lead to critical complications in the absence of proper care to the wounded area.

The healing process of foot ulcers is complex and is also interrupted by local factors such as moisture, infection, and the dressing method along with systemic factors such as age and nutritional status. Effective management of DFU starts with physical examination and selection of an appropriate wound care intervention. The focus is on achieving the goal of rapid and complete wound healing. In the holistic approach of wound management, three main areas addressed are tissue loss, ischemia, and infection [7]. In addition, the debridement of the wound is done regularly to keep it free of nonviable tissue. Suitable dressings are used to reduce the risk of infection, improve outcomes by controlling bacteria in the wound, and provide an optimal healing environment.

Wound irrigation is one of the essential components of wound management. It is the single greatest intervention in wound care that can reduce the risk of infection. The goal of wound irrigation is to remove foreign material, decrease bacterial contamination of the wound, and to remove cellular debris or exudate from the surface of the wound. Irrigation stimulates neovascularization and healthy cell proliferation. Common irrigation solutions used for wound irrigation are normal saline, sterile water, or potable water when normal saline or sterile water is not available [8]. Some compounds such as hydrogen peroxide, eusol, and collagenase ointment are also used for wound healing [9].

**Figure 1:**
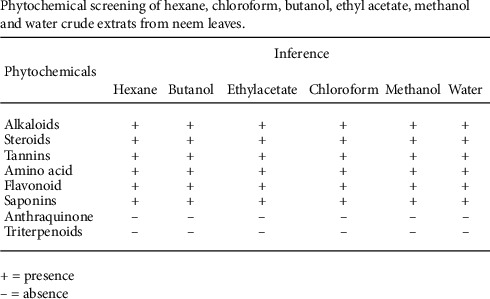
Phytochemical screening of hexane, chloroform, butanol, ethyl acetate, methanol, and water crude extracts from neem leaves.

Using plants with medicinal properties to treat wounds have been found useful in fighting against infection and accelerate wound healing [10–12]. The fruiting branches of Choluteca and Cilicia Boiss. and Bal., along with the leaves and fruits, have been used to heal inflammatory wounds as traditional medicine in various parts of Turkey [13]. Many medicinal plants have antioxidant and antibacterial properties. *Azadirachta indica* (neem), a Meliaceae family tree, has been used in India for many years in the treatment of several diseases in medicine and dentistry. Phytochemical screening of neem leaf showed (Figure 1) the presence of medicinal properties. Almost all parts of the neem plant are used for medicinal purposes and for the treatment of inflammation, infections, fever, skin diseases, dental problems, and diabetes. Neem extract contains flavonoids, alkaloids, steroids, saponins, and tannins [14]. Few studies showed that neem oil could be used for chronic nonhealing wounds.

Most of the population in many developing countries relies on traditional medicine for their everyday health needs, often integrating it on an adhoc basis with modern medicines [15]. Individuals with chronic wounds often experience significant financial burden due to prolonged periods of treatment which require dressings. Improving healthcare and making affordable health services available are one of the most important challenges for nurses. Due to the escalating cost of healthcare especially in wound management and location of health center at distant places, it is economical to use herbal plant extracts to treat chronic wounds. Expanding research on the use of medicinal plants in wound management is found to be very essential. Considerable amount of clinical research on using neem extract for irrigation of wounds has been conducted and published. However, such studies have been performed on animals and irrigation for dental treatment. In India, neem leaves are considered important and are used in traditional medicine, especially in dentistry. It is also a proven fact that neem has various pharmacological uses such as antibacterial, antiallergic, antifungal, and medicinal treatment [16]. It is a known fact that normal saline wound irrigation is the conventional practice of wound management and has been followed for many decades. There is paucity of information on the effect of aqueous neem leaf extract used for chronic wounds especially for diabetic foot ulcers. Reviewing the importance and use of neem leaves in national, regional, and international perspective is needed to study the effect of neem extract irrigation practice for better economic and therapeutic utilization on the wound healing in diabetic foot ulcers.

The purpose of this study was to evaluate and compare the efficacy of neem leaves extract with normal saline irrigation practice in wound dressing on healing outcome in diabetic foot ulcers. The objectives of the study were (a) to assess and compare the effectiveness of neem leaves extract irrigation to normal saline irrigation practices on wound healing outcome in diabetic foot ulcers and (b) to estimate and compare the effectiveness of neem leaves extract and normal saline irrigation practice on clinic-physiological parameters in individuals with diabetic foot ulcers.

## 2. Methods

### 2.1. Study Design

**Figure 2:**
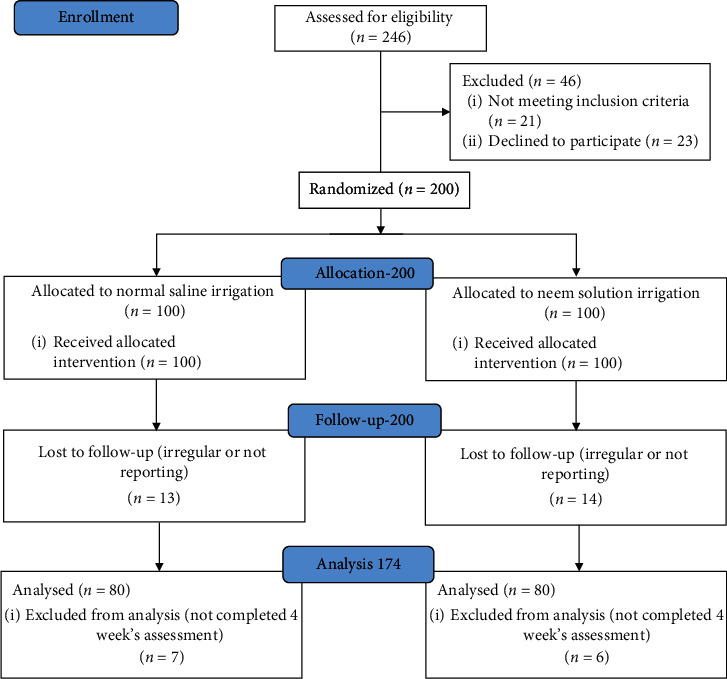
The research process.

A quantitative research approach was used to compare the effectiveness of normal saline and neem leaf extract irrigation on wound healing parameter and clinic-physiological of DFU individuals. A quasiexperiment with a repeated measures design was used on two study groups (Figure 2). One group received neem leaf extract wound irrigation and another group received normal saline wound irrigation.

### 2.2. Setting and Samples

This study was conducted among individuals with diabetic foot ulcer who attended a foot care clinic at a private hospital in Assam, India. Based on the pilot study effect size, the minimum sample size required was 80 per group. To increase the credibility, random allocation of 100 participants to each group was performed using the simple random sampling technique. The study included individuals who (i) were minimum 20 years of age, (ii) were diagnosed as diabetic with a foot ulcer of any duration, (iii) had a wound with an area of at least 1 cm^2^ (greatest length greatest width), (iv) had foot ulcers of Gr 1–3 as per Wagner classification, (v) were uncomplicated by clinical signs of severe ischemia, (vi) had the support of caretakers to adhere to nonweight bearing practices, and (vii) were hemodynamically stable. The study excluded individuals (i) with DFU of grade 4 or 5 as per Wagner classification, (ii) had drug problems or alcoholics, (iii) had any cognitive or mental illness, (iv) had any prior hospitalization for DFU, and (v) were under treatment on immunosuppressive drugs or on treatment for end stage renal disease.

### 2.3. Instruments/Measurements

#### 2.3.1. Sociodemographic Information Sheet

The demographic and clinical variables of all DFU individuals was included and consisted of age, gender, education, occupation, monthly income, lifestyle habits on alcohol and smoking, history, and awareness of self-footcare practices and diet control practices.

#### 2.3.2. Clinical Information Sheet

The data included type and duration of diabetes, medication for diabetes, precipitated event of foot ulcer, presence of peripheral pulses in the affected limb, foot deformity, vibration sensation, limb color, and the skin and nail condition of the affected limb. Wagner classification was followed for grading the severity of the foot ulcers. As per the classification, grading was (i) Grade 0, no open lesions and may have deformity or cellulitis, (ii) Grade 1, localized superficial ulcer, (iii) Grade 2, ulcer extension to ligament, tendon joint capsule, or deep fascia, without abscess or osteomyelitis, (iv) Grade 3, deep ulcer with abscess, osteomyelitis, or joint sepsis, (v) Grade 4, gangrene localized to portions of toes, and (vi) Grade 5, extensive gangrenous involvement of the entire foot. Infection of foot ulcer were checked and classified as per the guidelines [17] given by the Infectious Diseases Society of America (IDSA) and International Working Group on the Diabetic Foot (IWGDF). The ulcer was graded as uninfected, mild infection, moderate infection, and severe infection with scores of 1, 2, 3, and 4, respectively.

#### 2.3.3. Clinic-Physiological Parameters Measurement

This included assessment of parameters such as temperature, pulse rate, respiration, blood pressure, wound pain, local warmth, random blood sugar, and HbA1C measurement.

#### 2.3.4. Wound Score (PUSH Score) Measurement

The pressure ulcer scale for healing (PUSH) tool [18] was used to measure the status of the foot ulcers of the individuals. It is fast and reliable. The PUSH tool measured three parameters and subscales which are considered most indicative of healing–(i) wound size (greatest length *×* greatest width = wound surface area), (ii) exudate amount (estimated as light, moderate, or heavy after removal of the dressing), and (iii) tissue type (closed, epithelial tissue, granulation tissue, slough, and necrotic tissue/eschar). The subscale scores generated the total score, which ranged from 0 to 17. Higher scores indicated worse ulcer conditions and diminishing scores indicated improvement in the wound healing process.

### 2.4. Interventions

Group 1 received wound care with normal saline irrigation twice a week for four weeks, while Group 2 received wound care with freshly prepared neem leaf (leaves identified and issued specimen voucher by an authorized officer of the Assam Agriculture University) extract irrigation twice a week for four weeks. On the first day of the enrolment for the study, preintervention wound assessment was performed using the PUSH tool on the wound size, type of tissue on wound bed, and amount of exudate. Wounds which required debridement were treated by the treating surgeon according to standard procedures. The wound was irrigated with normal saline for group 1 and by freshly prepared neem leaves extract solution in group 2. For both groups, irrigation was performed using a 10 ml syringe with 19-gauge needle following the aseptic procedure technique. Subsequently topical antibiotics (Metrogyl-P and Metrogyl gel) were applied to the wound. The wound was finally dressed with a dressing by gel or foam and covered with dressing material. A secondary dressing was allowed to add cushion for support. Offloading was performed using a cast walker, or diabetic shoe, or wheelchair which was required for all participants in both groups.

### 2.5. Data Collection Procedure

Data were collected from February 01, 2019, to February 28, 2020. Individuals with diabetic foot ulcers who met the inclusion criteria were randomly assigned into two groups: normal saline and neem leaf extract groups. Clinic-physiological parameters (temperature, pulse rate, respiration rate, blood pressure, wound pain, and clinical infection of wound) and preintervention wound assessment was performed on the initial day of visit. After the initial wound assessment, intervention wound irrigation and wound care was given to both groups. All participants reported to the foot clinic every third or fourth day for follow-up. Postintervention wound assessment and clinic physiological parameters were carried out at the end of every week till the completion of four weeks of intervention. Wounds which required debridement were performed by the treating surgeon. Wound culture was taken from infected wounds from both groups and treated with antibiotics as prescribed by the treating physician.

### 2.6. Data Analysis

Demographic variables were summarized as frequencies and percentages. The Shapiro–Wilk test was used to verify the normal distribution of the observed data. *F*2 tests were used to verify the homogeneity of the demographic and clinical variables. One-way ANOVA was used to verify the homogeneity of the dependent variables. Repeated measures ANOVA was used to find out the effectiveness of the intervention on the dependent variables. Post hoc pairwise comparisons were performed with the Bonferroni test. The analysis was carried out using SigmaPlot software, and *p* values smaller than 0.05 were considered statistically significant.

### 2.7. Ethical Considerations

The study protocol was approved by the Regional Ethical Committee registered with CDSCO, Government of India, vide Regd. No. ECR/487/Inst/AS/2013/RR-16 dated 19 Feb 2019. The study was conducted in accordance with the code of ethics of the Declaration of Helsinki and all its amendments. The data were collected after obtaining written informed consent from all participants of the study. Purpose of the study and right to withdraw from study at any point was explained to all participants. Privacy and confidentiality of the participants was maintained throughout the study. Plant leaves (neem) were identified by an authorized official of Assam Agricultural University, India, and a specimen voucher was issued for this study.

## 3. Results

The demographic and clinical characteristics of the participants were recorded. The chi square test was carried out to find out the homogeneity of the participants, and all demographic (Table 1) characteristics were homogeneous (*p* > 0.05). Homogeneity of the wound variables in both the groups were determined by one-way ANOVA and were found to be homogenous (*p* > 0.05), with regards to age of the wound and wound surface area (Table 2).

The average of the wound mean score in the normal saline group and neem extract group was significantly reduced from 12.93 to 9.14 and from 11.97 to 6.7, respectively (*p* < 0.001). Bonferroni's pairwise comparison (Table 3) revealed a significant difference in wound score reduction between normal saline and neem extract groups (*p* < 0.001). The results of repeated measures ANOVA were also significant (*p* < 0.001) for the wound score reduction (Table 4).

The mean wound area decreased from 19.84 ± 23.97 cm^2^ to 14.31 ± 21.43 cm^2^ in the normal saline group (*p* < 0.03) and 17.14 ± 21.45 cm^2^ to 7.04 ± 16.72 cm^2^ in the neem leaf group (*p* < 0.001). Similarly, wound area scores also reduced significantly in both groups, normal saline (*p* < 0.005) and neem leaf extract (*p* < 0.001). The mean preintervention exudate score in the normal saline group and the neem leaf group was 1.93 ± 0.244 and 1.563 ± 0.499, respectively. The mean exudate scores postintervention at the end of week four in the normal saline group and the neem leaf group were 1.288 ± 0.637 and 0.918 ± 0.458, respectively. We found that this was statistically significant (*p* < 0.001) on the posttest in both groups. The mean preintervention tissue scores in the normal saline group decreased from 2.738 to 1.877 and from 2.550 to 1.689 in the neem leaves extract group, at the end of four weeks.

We compared the of percentage wound area reduction (PWAR) and percentage wound score reduction (PWSR) in saline and neem groups at the end of four weeks of intervention. Result showed that in the neem group, 82.3% (51/62) individuals achieved ≥50% PWAR when compared to 55.8% (29/52) individuals in the saline group, while 17.7% (11/62) individuals in the neem group and 44.2% (23/52) in the saline group achieved <50% PWAR. The *F*2-analysis revealed that both groups differed significantly (*p*=0.002).

Results showed that in the neem group, 80.6% (50/62) individuals achieved ≥50% PWSR when compared to 55.8% (29/52) individuals in the saline group. It was observed that 19.4% (12/62) individuals in the neem group and 44.2% (23/52) in the saline group achieved <50% PWSR. The F2 analysis revealed that both groups differed significantly (*p*=0.004). Correlation of HbA1C with wound score in pretest day one and posttest at the end of week four was studied. The Pearson correlation test revealed a weak correlation between HbA1C and wound score in both groups at baseline (*r* = 0.272, *p*=0.0428) and after week four (*r* = 0.190, *p*=0.0428). Our findings suggested that the control of the sugar level promoted wound healing in diabetic foot ulcers.

The results of two-way repeated measures ANOVA (Table 5) revealed no significant differences between systolic blood pressure (SBP), diastolic blood pressure (DBP), body temperature, pulse beat, respiration, wound pain, and local warmth (*p* > 0.05) in both groups. While for clinical infection grade, though the observed difference was clinically significant, the result was not statistically significant (*p*=0.05).

## 4. Discussion

The aim of the current study was to compare the effectiveness of normal saline and neem leaf extract irrigation on wound healing of diabetic foot ulcers. The findings have revealed that there is a significant effect of neem irrigation on the wound healing among the diabetic foot ulcer participants. Significant reduction in wound area and wound score was achieved among individuals with foot ulcer in the neem extract group compared to the normal saline group. Jaya Mary and others (2017) compared the effectiveness of conventional and herbal treatment in diabetic foot ulcer patients, and their results showed a highly significant change in the third visit after 30 days of the posttest (*p* < 0.001). In the aforementioned study, the dressing was carried out with a mixture of herbal oil prepared with fresh dark green neem (*Azadirachta indica*) leaves, coconut oil (*Cocos nucifera*), and turmeric powder (*Curcuma longa*) [19]. Iabichella et al (2013) noted in a clinical case that fusion of two plant extracts, Hypericum (*Hypericum perforatum*) and neem oil, helped decrease the dimension of the ulcer and increased the granulated tissue and remodeling the skin tissue in the lesion. In a different study, the same researchers found an improvement of peripheral microvascular circulation in neuropathic patients with advanced DFU using the same extract [20].

It is proved that leaf extract of neem has antimicrobial activity against human pathogenic bacteria [21]. In another study, wound dressing prepared with extract from natural sources of neem leaves and turmeric was evaluated for the efficacy of the product by various physical and biochemical tests [22]. Jamshidi et al. (2018) reported that medicinal plants were important sources of new chemical substances that have beneficial therapeutic effects [23]. It is assumed that ingredients from medicinal plants are less toxic and have fewer side effects compared with orthodox therapeutic agents. In the present study too, patients who underwent neem extract irrigation did not experience any kind of reactions or adverse events. Analysis of the present study also revealed that both normal saline and neem leaf extract irrigation practice during wound dressing made no significant changes to body temperature, pulse rate, respiration rate, blood pressure, local warmth, and wound pain in both groups. Similarly, Jaya Mary and others (2017) reported no significant change in systolic and diastolic blood pressure both in conventional treatment and herbal treatment [19]. When comparing the saline group, there was a reduction in the infection grade in the neem group; however, it was not significant (*p*=0.053). These finding suggests that the neem leaf extract irrigation during dressing for DFU individuals is safe and effective in controlling the clinical infection.

In a Thailand study, authors found that approximately 56.8% of DFU patients had neuropathy, while in another study, 43.75% had any one of the neuropathy symptoms such as numbness, pain, or loss of vibration sensation [24]. Altered vibratory sensation was observed in 42.5% among both groups in this study, while a study by Parisi et al. (2016) demonstrated a statistically significant association with the risk of ulceration and amputation of the feet of diabetes mellitus patients [25]. Almost all study participants were unaware about the self-foot care practices, while 48.75% did not know about the cause of their foot ulcers. Coexistence of neuropathy along with lack of foot care is the main cause of the tendency for progression of their lesions before presentation [26].

Literature reveals that healing of wounds is affected by many factors. However, none have been consistently identified as an early predictor of wound healing [27]. In the current study, the mean reduction in the ulcer area at four weeks was 1.5 cm^2^ in the neem leaf extract group versus 0.8 cm^2^ in the normal saline group (*p*=0.047). Overall, in both groups, the number of individuals in whom ulcers achieved ≥50% wound area reduction (PWAR) was more in the neem leaves group (64%) compared to 35% in the normal saline group during the four-week study period. This indicated the effect of the neem leaves irrigation on the ability of the 4-week changes in an ulcer area. Ajaz et al. (2015) reported in their study that the average size of ulcer surface area was 13.3 ± 2.1 cm^2^ on the first visit of the subjects prior to start of the therapy was reduced to 3.1 ± 0.7 cm^2^ after 20 days of topical application of Amp care (oil-based formulation made out of extracts of bark of *Azadirachta indica*, rhizome of *Curcuma longa*, and leaves of *Trichosanthes dioica*) [28]. There are bioactive substances with antiseptic and anti-inflammatory properties in some plants and hence are able to heal wounds [29]. The effects of medicinal plants on wound healing may be linked to the free radical scavenging action of compounds in the extracts acting either singly or synergistically [30]. Wong et al. (2001) reported in their study that amputation could be avoided in 87% of diabetic wound using Chinese herbal medicine [31]. In one particular case, the application of an ointment derived from medicinal plants prevented 85% of infected diabetic wound from the amputation of their legs [28].

We believe that neem leaves extract irrigation might actually add the synergistic antimicrobial effect without causing advert drug interaction. Ineffectiveness of neem extract on all patients in achieving ≥50% PWAR at four weeks of time might be due to the presentation of individuals with higher grade of ulcers and age of wound and could be due to other confounding factors such as nutritional factors. It is found through this study that neem leaves extract solution can easily be prepared at home and can be used to manage foot ulcers at home. To establish a holistic approach in the evidence-based wound management by the nurse practitioners, the present study indicates the demand for developing alternative methods for managing DFU using plant extracts that have significant wound healing properties.

This study has a few limitations. First, the neem leaves extract having antimicrobial properties was only studied for Wagner's Grade I to Grade III ulcers. Second, we had a smaller sample size. Hence, the results cannot be generalized. However, for future research, using neem leaves extract solution for DFU dressing could be studied for more samples selected randomly and using the double blind method in community setting.

## 5. Conclusion

Managing DFU using neem extract solution proves to be an effective measure in healing the ulcer. Reduction of wound healing score (PUSH score) and other wound variables such as wound area, tissue type, and wound exudate improved significantly in the neem leaves extract group when compared with traditional normal saline wound irrigation. This study concludes that neem extract solution is promising, easy to prepare, and forms a natural wound care management for DFU. We also found that this solution is very safe as it did not cause any complications systematically during the four weeks of the study. Managing DFU requires continuous foot care and early risk identification of ulcer.

## Figures and Tables

**Table 1:** Demographic and clinical characteristics of diabetic foot ulcer individuals at baseline.

Age (yr)	21–40	4 (5)	9 (11)	2.281	0.320
	41–60	51 (64)	50 (63)		
	61–80	25 (31)	21 (26)		

Gender	Male	68 (85)	64 (80)	0.693	0.405
	Female	12 (15)	16 (20)		

Marital status	Married	78 (98)	78 (98)	0.000	1.000
	Single	2 (2)	2 (2)		

Education	Postgraduate	5 (7)	4 (6)	0.107	0.744
	Graduate	23 (29)	19 (24)		
	Secondary	6 (8)	9 (10)		
	Primary	45 (56)	48 (60)		

Occupation	Employed	58 (73)	59 (74)	0.032	0.858
	Unemployed	22 (27)	21 (27)		

Type of family	Joint	31 (39)	29 (36)	0.107	0.744
	Nuclear	49 (62)	51 (64)		

Monthly income (Indian rupees)	<20,000	31 (39)	47 (59)	6.831	0.77
	21,000–40,000	33 (41)	20 (25)		
	41,000–60,000	14 (17)	11 (13)		
	61,000–80,000	2 (3)	2 (3)		

Duration of DM	<5 years	23 (29)	29 (36)	4.564	0.335
	5–15 years	26 (32)	28 (35)		
	15–25 years	20 (25)	18 (23)		
	>25 years	11 (14)	5 (6)		

HbA1c level	≤7	6 (8)	8 (9)	0.08	0.772
	>7	74 (92)	73 (91)		

Vibration sensation	Present	34 (43)	36 (45)	0.626	0.744
	Absent	46 (57)	44 (55)		

NS, normal saline; NE, neem leaves extract irrigation; DM, diabetes mellitus.

**Table 2:** Wound characteristics of diabetic foot ulcer individuals at baseline.

Wound variables	Groups		*F* ^2^	*p*
	NS (*n* = 80)	NE (*n* = 80)		
	*n* (%)	*n* (%)		
Age of wound (days)				
<100	74 (93)	74 (93)	0.533	0.912
101–200	3 (3)	2 (2)		
201–300	1 (1)	2 (2)		
>300	2 (3)	2 (3)		

Ulcer grade				
Grade I	3 (4)	19 (24)	27.321	0.001
Grade II	38 (47)	49 (61)		
Grade III	39 (49)	12 (15)		

Wound area (Sq. cm)				
<10	30 (38)	36 (45)	3.52	0.318
10–50	43 (54)	40 (50)		
51–90	5 (2)	1 (1)		
>90	2 (3)	3 (4)		

Tissue type				
Granulation	36 (45)	21 (27)	6.13	0.013
Slough	44 (55)	59 (74)		

Exudate level				
Moderate	0 (0)	20 (25)	22.56	<0.001
High	80 (100)	60 (75)		

Infection grade				
Nil	1 (1)	13 (16)	23.976	0.001
Mild	55 (69)	27 (34)		
Moderate	24 (30)	40 (50)		

NS, normal saline; NE, neem leaves extract irrigation; DM, diabetes mellitus.

**Table 3:** Mean PUSH scores with Bonferroni-adjusted pairwise *t* tests in normal saline and neem leaf extract groups.

Time	Groups	Mean	Diff. in means	*t*	*p*
Baseline	NS	12.937	0.963	2.161	0.032
	NE	11.975			

Week 1	NS	12.387	1.475	3.312	0.001
	NE	10.912			

Week 2	NS	11.287	1.775	3.986	<0.001
	NE	9.512			

Week 3	NS	10.402	2.400	4.778	<0.001
	NE	8.002			

Week 4	NS	9.153	2.449	4.457	<0.001
	NE	6.704			

NS, normal saline; NE, neem leaves extract irrigation.

**Table 4:** Results of RM-ANOVA of PUSH score among normal saline and neem leaf extract groups.

Group factor	1	553.816	553.816	20.278	<0.001

Time factor	4	1690.558	422.640	221.685	<0.001

Interaction of group and time	4	52.651	13.163	6.904	<0.001

**Table 5:** Mean, SEM, and RM-ANOVA of clinicophysiological parameters among normal saline (NS) and neem leaves extract (NE) groups.

Variable	Groups	Time	Mean ± SEM	Source	*f*	*p*
SBP	NS	Pretest	131.625 ± 1.769	Time Group	0.119 Df = 4	0.976
		PO Wk-4	125.750 ± 1.769			
	NE	Pretest	127.625 ± 1.769			
		Week 4	121.250 ± 1.769			

DBP	NS	Pretest	82.000 ± 0.838	Time Group	0.814 Df = 4	0.516
		PO Wk-4	79.250 ± 0.838			
	NE	Pretest	80.250 ± 0.838			
		PO Wk-4	77.500 ± 0.838			

Temp	NS	Pretest	98.613 ± 0.331	Time Group	0.783 Df = 4	0.537
		PO Wk-4	98.670 ± 0.331			
	NE	Pretest	98.580 ± 0.331			
		PO Wk-4	98.678 ± 0.331			

Pulse	NS	Pretest	82.550 ± 0.368	Time Group	2.714 Df = 4	0.007^*∗*^
		PO Wk-4	81.925 ± 0.368			
	NE	Pretest	81.275 ± 0.368			
		PO Wk-4	81.075 ± 0.368			

Respiration	NS	Pretest	17.700 ± 0.125	Time Group	1.292 Df = 4	0.272
		PO Wk-4	17.325 ± 0.125			
	NE	Pretest	17.850 ± 0.125			
		PO Wk-4	17.575 ± 0.125			

Wound pain	NS	Pretest	1.863 ± 0.023	Time Group	0.935 Df = 4	0.443
		PO Wk-4	1.720 ± 0.030			
	NE	Pretest	1.825 ± 0.023			
		PO Wk-4	1.654 ± 0.027			

Clinical infection grade	NS	Pretest	2.337 ± 0.007	Time Group	2.354 Df = 4	0.053
		PO Wk-4	2.337 ± 0.005			
	NE	Pretest	2.287 ± 0.006			
		PO Wk-4	2.252 ± 0.005			

SBP, systolic BP; DBP, diastolic BP; PO Wk-4, postintervention at week 4.

## Data Availability

The data used to support the findings of this study are available from the corresponding author upon request.
